# Spatial distribution of the active surveillance of sheep scrapie in Great Britain: an exploratory analysis

**DOI:** 10.1186/1746-6148-5-23

**Published:** 2009-07-16

**Authors:** Colin PD Birch, Ambrose C Chikukwa, Kieran Hyder, Victor J Del Rio Vilas

**Affiliations:** 1 Veterinary Laboratories Agency – Weybridge, New Haw, Addlestone, Surrey, KT15 3NB, UK; 2 Department for Environment, Food and Rural Affairs (Defra), Nobel House, 17 Smith Square, London, SW1P 3JR, UK

## Abstract

**Background:**

This paper explores the spatial distribution of sampling within the active surveillance of sheep scrapie in Great Britain. We investigated the geographic distribution of the birth holdings of sheep sampled for scrapie during 2002 – 2005, including samples taken in abattoir surveys (c. 83,100) and from sheep that died in the field ("fallen stock", c. 14,600). We mapped the birth holdings by county and calculated the sampling rate, defined as the proportion of the holdings in each county sampled by the surveys. The Moran index was used to estimate the global spatial autocorrelation across Great Britain. The contributions of each county to the global Moran index were analysed by a local indicator of spatial autocorrelation (LISA).

**Results:**

The sampling rate differed among counties in both surveys, which affected the distribution of detected cases of scrapie. Within each survey, the county sampling rates in different years were positively correlated during 2002–2005, with the abattoir survey being more strongly autocorrelated through time than the fallen stock survey. In the abattoir survey, spatial indices indicated that sampling rates in neighbouring counties tended to be similar, with few significant contrasts. Sampling rates were strongly correlated with sheep density, being highest in Wales, Southwest England and Northern England. This relationship with sheep density accounted for over 80% of the variation in sampling rate among counties. In the fallen stock survey, sampling rates in neighbouring counties tended to be different, with more statistically significant contrasts. The fallen stock survey also included a larger proportion of holdings providing many samples.

**Conclusion:**

Sampling will continue to be uneven unless action is taken to make it more uniform, if more uniform sampling becomes a target. Alternatively, analyses of scrapie occurrence in these datasets can take account of the distribution of sampling. Combining the surveys only partially reduces uneven sampling. Adjusting the distribution of sampling between abattoirs to reduce the bias in favour of regions with high sheep densities could probably achieve more even sampling. However, any adjustment of sampling should take account of the current understanding of the distribution of scrapie cases, which will be improved by further analysis of this dataset.

## Background

Since 2002, the European Union has required that each Member State must test a representative sample of its sheep population to monitor scrapie prevalence [1]. In Great Britain, during the period 2002–2005, this active surveillance on sheep older than 18 months included samples taken in abattoir surveys (AS, c. 132,000) [2] and from sheep that died in the field (fallen stock (FS), c. 19,600) [3]. These samples are used for surveillance of both classical and atypical scrapie, which are distinct prion diseases of sheep. Classical scrapie has been recorded in Britain for over 200 years, while atypical scrapie was only recently recognized, but has probably existed for a long time [4]. With respect to surveillance, the main difference between the diseases is that sheep with atypical scrapie develop clinical symptoms and die older than sheep with classical scrapie, so that they are more likely to survive to an age at which they are sent to abattoir as mature animals [5]. Of the two surveys, the abattoir survey is closer to a random sample, because selection at abattoirs is potentially random and farmers do not know which, if any of their sheep will be sampled. The fallen stock survey provides a higher proportion of samples positive for classical scrapie [6].

The Animal Movements Licensing System (AMLS) in England and Wales has recorded all movements of batches of sheep and other animals, including movements from Scotland, since 2001, being kept as a digital database (AMLS2) since 2005. Recent tracing of birth holdings using AMLS2 [7] provided the opportunity to evaluate the "representativeness" attribute of the active component of the scrapie surveillance [8]. Most directly, tracing the holdings of origin allowed evaluation of the spatial distribution of sampling.

There have been several studies of spatial patterns of scrapie reporting and disease in Great Britain [9-12]. All these studies assessed the spatial distribution either on data from postal surveys, which had low spatial resolution, or from the statutory reporting of clinical cases, which had unknown self-reporting bias. The active surveillance data offers high spatial resolution and more opportunities to analyse the distribution of sampling. Spatial analysis of sampling may allow the detection of under-sampling or over-sampling at the spatial unit of choice. Local sampling intensities can also be used to correctly estimate the local prevalence estimates obtained from surveys [4]. Studies in France have already demonstrated that the design and geographic distribution of sampling surveys could bias their results [13,14].

Although the pitfalls of spatial visualization have long been recognized [15,16], it is widely acknowledged for its value in exploration and analysis of spatial data [17]. An example is the use of area cartograms, which are particularly well suited to displaying sampling rates and denominator populations together. A cartogram is a map transformed so that regional areas are proportional to a measure of interest (i.e. the number of sheep holdings in a county), rather than actual land area [18]. We used this method to emphasize counties according to their weight in the sheep population, while still making them readily recognizable. A further advantage was that the cartograms could display more information, for example the number of holdings was represented by county area, while sampling rate was displayed by use of colour scales.

Our primary goal was to assess whether sampling in Great Britain was uniformly distributed in space relative to the population of sheep holdings. Uniform sampling rate would contribute to achieving representative sampling as required by European legislation, and would help against failing to detect clusters of infection that coincided with areas with low sampling rate. We achieved this primary goal through visualization, by mapping county sampling rates. Having demonstrated uneven sampling, we made a preliminary spatial analysis to assess overall and local spatial and temporal autocorrelation, and to investigate apparent correlations between sampling rate and sheep density.

## Methods

### Numerator data

We traced birth holdings by comparing flock marks from identity tags of sampled sheep with flock marks recorded against sheep movements in the Animal Movements Licensing System (AMLS2) database in the period January 2005 to February 2006 inclusive. For each movement record, AMLS2 records the flock marks of the sheep being moved and the identity of the holding they are leaving. The birth holding for a flock mark was identified as the holding most frequently recorded as moving off sheep with that flock mark. Comparison of traces known to be reliable from corroborating evidence with traces of unknown reliability allowed definition of criteria by which traces were selected to increase reliability. These criteria were based on the number of departures of a flock tag from the presumed birth holding and the number of departures from other holdings. This technique was called 'shotgun' tracing [7]. Scottish Animal Movement System (SAMS) data was not used because of problems with the availability of flock mark data. This meant that we could not use data on movements wholly within Scotland, which may have reduced tracing of Scottish holdings, but we had the opportunity to check the impact of tracing on the denominator population, as explained below.

### Denominator data

We used two sources of denominator data at the holding level. The first was the 2004 June Agricultural Survey of England, Scotland and Wales (referred to here as the 'agricultural survey') and the second was the shotgun tracing. The agricultural survey is probably the most accurate source of holding level data currently available, but involves a degree of sampling and interpolation. The alternative was to use the criteria for selecting traces in the shotgun method to generate a list of all holdings that could be traced as birth holdings of sampled sheep. The list of traceable holdings was the appropriate denominator population, because it included all holdings that might be identified as sampled and no others. To check regional bias introduced by tracing, we compared the number of holdings on the list of traceable holdings against the number in the agricultural survey in each county, by using a scatter plot and calculating their correlation.

### Locations

Identifying locations for veterinary records and samples is not a trivial problem [19]. From the AMLS database we were able to obtain a county parish holding ID (CPH), with postcode, map reference, or easting and northing coordinates for each traced holding. Usually the location was for a mailbox in a farmhouse associated with a particular group of sheep, so it would be close to the animals, although probably not in the centre of their grazing area. Each location was checked against the parish identified by the first five digits of the CPH. If the location was outside the parish, it was corrected to the parish centroid. Thus every holding was located within the parish and therefore the county identified by its CPH.

### Sampled proportions and cartograms

The unit for control of scrapie is the farm or holding, so we focused our analyses on the proportion of holdings sampled within each county (holding sampling rate). The holding sampling rate was the number of holdings sampled in a given county divided by the number of traceable sheep holdings in the county. Sampling rates were calculated for the AS and FS surveys separately, and combined. However, in case sampling was more closely related to the number of sheep in a region, rather than the number of holdings, maps for 2002–2005 were also prepared to show the number of samples from each county as a proportion of the county's adult sheep population. Agricultural survey information was used to provide the denominator population for these maps, because it was the only source for numbers of sheep.

All maps were produced using ArcMap . Most maps were cartograms, which were transformed so that the area of each county was proportional to the number of holdings in it, using the CartogramCreator script , which applies a "rubber sheet" method [20,21]. The maps of proportions of sheep sampled were also used to display the locations of abattoirs used for sampling, so they were presented as regular land area based maps.

### Spatio-temporal correlation

Uneven sample distribution among counties could result from temporary and/or local causes, but broad regional trends that were consistent through time and space might have more impact and be easier to adjust. We therefore assessed whether there was evidence of spatial correlation in sampling between counties across Great Britain and of temporal correlation between years. Because global measures tend to conceal local variation, we also looked for individual counties with significantly high or low sampling rates, taking account of the trend in their neighbourhoods.

To test for consistency in time, we calculated the correlation matrix for the county sampling rates between different years, comparing within surveys and between them. We measured global spatial autocorrelation across Great Britain using Moran's Index [22].



where *n *= the number of counties;  = the mean of the sampling rate across all counties; *x*_*i*_, *x*_*j *_= sampling rates in counties *i *and *j *respectively; *w*_*ij *_∈ *W *= the weighting for the covariance between counties *i *and *j*; finally σ(x) = the sampling rate standard deviation.

The weight matrix *W *defines the neighbourhood structure in accordance with Tobler's First Law [23] by which measures at locations close to each other will tend to be more similar than measures at distant locations. We set elements of the weight matrix, *w*_*ij *_to be inversely proportional to the distance between the centroids of counties *i *and *j*. This weighting avoided assigning islands zero weights because they had no adjacent neighbours, which would have occurred if we had used a common alternative by which elements of the weight matrix would equal one if counties shared a boundary and zero otherwise. In addition, the elements are normalized so that row totals equal one. Our null hypothesis for testing the presence of spatial autocorrelation was that the observed sampling rates were randomly and independently assigned to counties. The expected value of Moran's *I*, *E *[*I*] = -1/(*n*-1) with *I *> -1/(*n*-1) indicating positive and I < indicating negative spatial autocorrelation.

The global Moran statistic tends to conceal local variation by assuming the absence of differences across a region [24,25]. To test for local clustering, we used a local version of the global Moran statistic, one of several measures known collectively as local indicators of spatial association (LISA) [26].



LISA statistics assess local associations by comparing local averages to global averages. The local statistic is large and positive when a county's sampling rate and the sampling rate in its neighbourhood are both substantially above the global average, or when both are substantially below the global average, which are termed "over-sampling" and "under-sampling clusters" respectively. The local Moran statistic is large and negative when a county's sampling rate is substantially above the global average while its neighbourhood's is substantially below average, or vice-versa, suggesting "outliers". Each county's local Moran statistic, *I*_*i *_is an indication of whether it is part of a local cluster or contrast with its neighbours.

Local and global statistics were calculated using Geoda , an open source spatial analysis system, and visualized on LISA cluster maps using ArcGIS . Both were compared to reference distributions that would be expected under the null hypothesis of no spatial correlation, which were generated randomly using Monte-Carlo simulations and a pseudo significance level [27].

### Distribution of sheep

A dilemma in the analysis of the distribution of sampling rates was the distinction between the sampling rate of sheep and the sampling rate of holdings. If sheep were individually selected at random at abattoirs, we would expect higher sampling rates from holdings with many sheep than holdings with few sheep. Therefore our analysis of sampling required consideration of the distribution of sheep as well as holdings. In addition to maps of the holding sampling rate, we mapped the distributions of sheep density and holding size (sheep per holding), as well as the sheep sampling rate. There were three measures of sheep density: areal sheep density (ha^-1^), holding density (km^-2^) and holding size (sheep/holding). Although these three measures were likely to be correlated with each other and were algebraically related, they were all identified as being potential factors influencing local sampling rate.

For both the AS and FS surveys, a generalized linear model with logit link was applied using Stata (Stata Corporation Ltd.) to model the number of holdings *u*_*i *_sampled during 2002–2005 in each county *i *containing *w*_*i *_holdings, as a binomially distributed variable related to the three measures of sheep density. The model was investigating the contribution of sheep density to local sampling rate, not attempting a complete explanation of variations in local sampling rate. Therefore significant deviation from the model fit was expected, so standard errors were scaled using the square root of the deviance-based dispersion. To assess the contribution of sheep density to the observed variance in sampling rate, squared deviations from the GLM model were calculated and compared with squared deviations from a null model that assumed the sampling rate *u*_*i*_*/w*_*i *_was constant.

## Results

### Tracing

Over the study period, 83,136 abattoir survey samples (63% of all AS samples) and 14,597 fallen stock samples (75% of all FS samples) were traced to 19,904 and 3,834 distinct holdings of birth respectively (Table 1). The majority of the sheep sampled as fallen stock died where they were born, so their birth holding was also the holding where they were collected [7]. Analysis of both surveys included all sampled sheep regardless of their suitability for scrapie testing.

**Table 1:** Number of holdings sampled each year.

	# Samples		# Selected samples		# Distinct holdings^1^	
	AS	FS	AS	FS	AS	FS
2002	31,865	1,066	20,609	730	8,818	541
2003	77,402	4,282	46,641	3,100	14,167	1,166
2004	11,041	5,018	7,380	3,918	4,378	1,333
2005	11,740	9,205	8,506	6,849	4,877	2,134

Total	132,046	19,571	83,136	14,597	19,904	3,834

^1^Totals are not simple sums because many holdings were sampled in more than one year.

**Figure 1:**
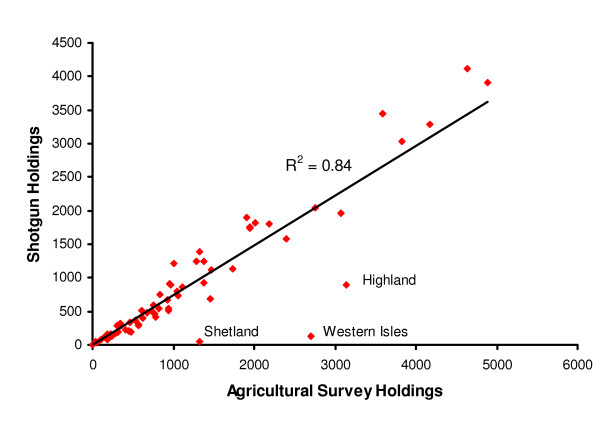
**Correlation between the number of traceable sheep holdings in each county and the number in the June 2004 agricultural survey**. Tracing used the "shotgun" method referred to in the text, applied to the Animal Movement and Licensing Scheme (AMLS) database. Outliers are labelled.

The total number of distinct sheep holdings in the 2004 agricultural survey of Great Britain was c. 80,000, while the total number of holdings that could be traced from AMLS using the new 'shotgun' method was c. 58,000 [7]. The numbers of holdings per county according to the two denominators (agricultural survey vs. 'shotgun') were strongly correlated (R^2 ^= 0.84, Figure 1). Most holdings in Shetland and the Western Isles and many in Highland could not be traced, because we could not use SAMS records of movements both starting and ending within Scotland, so this analysis effectively failed to include these areas. The scrapie active surveillance was known to take very few samples from Shetland or the Western Isles, so their omission from this study was acceptable. However, the proportions of traceable holdings in the rest of Scotland were as high as in England and Wales. Excluding the Scottish Highlands and Islands strengthened the correlation between numbers of traceable holdings and numbers of agricultural survey holdings in each county (R^2 ^= 0.96), providing reassurance that the tracing process had not introduced substantial geographical bias.

### Samples per holding

**Figure 2:**
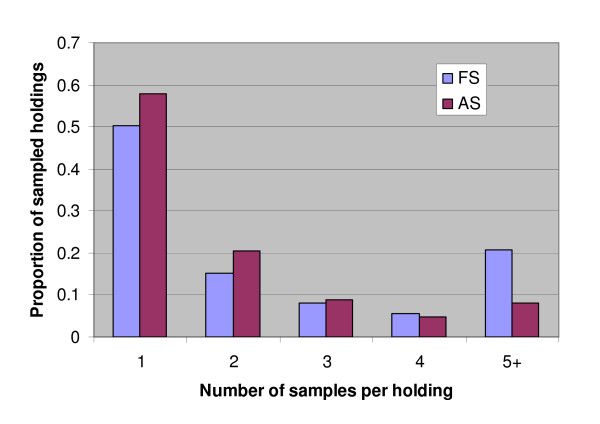
**Numbers of samples from sampled holdings in the fallen stock (FS) and abattoir (AS) surveys during 2004–2005**.

All 10,763 traced samples from the FS in 2004 and 2005 were compared with the 15,886 samples from the AS in the same years (Figure 2). Comparison was restricted to 2004 and 2005 because the numbers of samples from the two surveys in those two years were sufficiently close to be comparable. In the FS, 20.8% of holdings of birth provided 5 or more samples versus only 8.2% of AS holdings. The maximum number of samples from a single FS holding was 164 versus a maximum of 27 from any AS holding. The average number of samples per holding was 3.74 in the FS and only 2.08 in the AS. Thus the fallen stock survey included a substantial proportion of holdings providing many samples.

### Proportions of holdings sampled and case distribution

**Figure 3:**
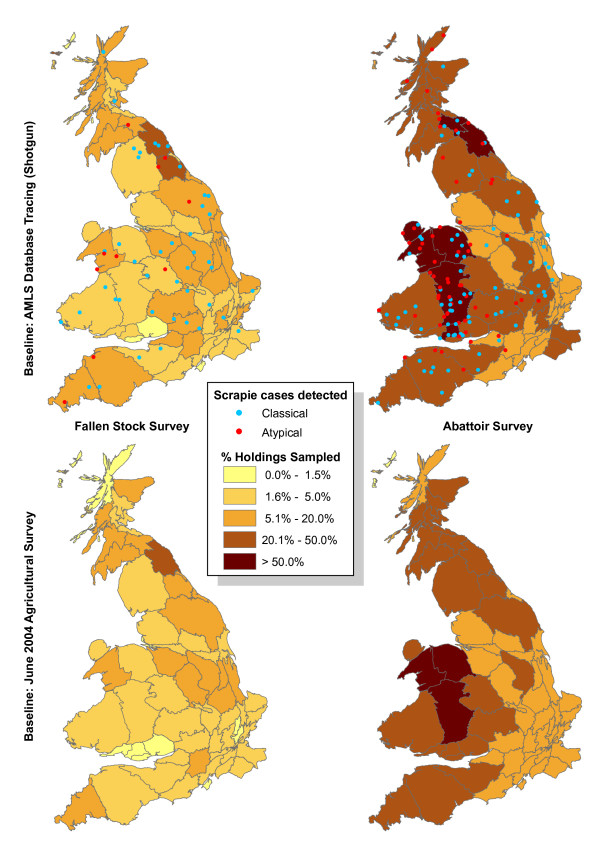
**Distribution of holding sampling rate in the fallen stock survey (left) and abattoir survey (right) during 2002 – 2005, with the scrapie cases detected**. The denominator sources are the 2004 agricultural survey (bottom) and traceable holdings from AMLS (top). Scrapie cases are not shown at their actual locations, but at random points within each county.

Sampling of holdings was clearly not uniformly distributed (Figure 3). The abattoir survey sampled the North and West of Great Britain, especially Wales, more intensely than the East. Areas sampled more intensely by the fallen stock survey seemed more localized, but tended to be more in the Midlands and East England, except for Gwynedd (North-west Wales). The choice of denominator (agricultural survey or 'shotgun') had little impact, but using the 'shotgun' denominator derived from AMLS avoided underestimating local sampling where the number of traceable holdings was relatively low compared with the number of agricultural survey holdings, e.g. in Highland, Fife, Kent and East Sussex. The population derived from the AMLS was used as the denominator for the rest of the analyses. The distribution of scrapie cases detected by active surveillance has been displayed to illustrate the impact of sample distribution on case distribution (Figure 3). As expected, local frequencies of scrapie detection are clearly correlated with local sampling intensities, especially in the abattoir survey. The difference between AS and FS in the relative number of cases of classical and atypical scrapie is related to the greater effectiveness of FS in detecting classical scrapie [6].

**Figure 4:**
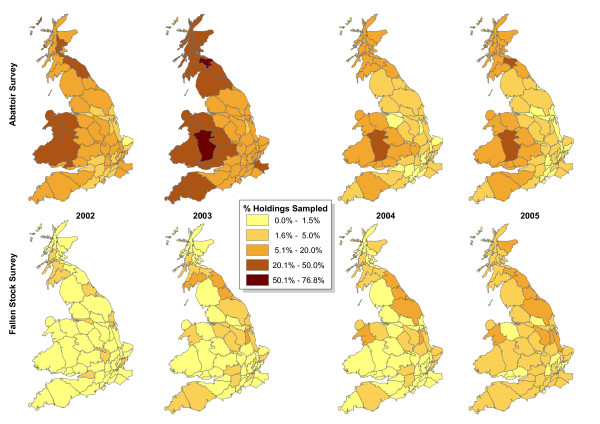
**Distribution of annual holding sampling rate during 2002 – 2005**.

Although the total numbers of samples differed substantially between years, the distribution of the sampling rate between counties (Figure 4) was similar each year and followed the overall distribution for 2002–2005 (Figure 3). Within each survey, sampling rates in different years were correlated; AS sampling was more strongly correlated between years than FS sampling (Table 2). The AS collected many more samples in 2002 and 2003 than in 2004 and 2005, while the FS collected more samples in 2004 and 2005 than the earlier years (Table 1). The differences between years in numbers of samples were due to the gradual development of the fallen stock sampling system, and changes in the sample quotas imposed by the European Union. The small number of FS samples in 2002 appeared sparse and scattered, matching the distribution in other years relatively weakly. The surveys were positively correlated with each other in 2004 and 2005, but correlations between surveys tended to be weaker than correlations within surveys (Table 2). Sampling from the two surveys combined was very similar to the abattoir survey alone in 2002 and 2003, because there were many more abattoir samples than fallen stock samples. In 2004 and 2005, combining the surveys reduced the relative standard deviation (RSD) among county sampling rates (Table 3). The RSD values also indicated that the variance of FS sampling rates among counties reduced from 2003 to 2005, a trend that is visible as an increasing number of counties with sampling rates falling in the range 1.6 – 5.0% in Figure 4.

**Table 2:** Correlation matrix for county sampling rates of sheep holdings for the abattoir and fallen stock surveys.

		Abattoir				Fallen Stock			
Abattoir	Year	2002	2003	2004	2005	2002	2003	2004	2005
	
	2002	1.000							
	2003	0.925	1.000						
	2004	0.827	0.703	1.000					
	2005	0.925	0.864	0.845	1.000				

Fallen Stock	2002	-0.026				1.000			
	2003		0.099			0.278	1.000		
	2004			0.234		0.100	0.728	1.000	
	2005				0.394	0.055	0.504	0.817	1.000

Correlation coefficients presented are within surveys between years and between surveys within years.

### Spatial Autocorrelation

At the national level, global Moran statistics for the AS indicated significant positive spatial autocorrelation (Table 3). In other words, counties with high AS sampling rates are likely to be close to other counties with high AS sampling rates and, conversely, counties with low AS rates are likely to be close to other counties with low AS rates. In contrast, although the RSD was of more or less similar magnitude, global Moran statistics did not indicate significant spatial correlation of FS sampling rates between counties. Global Moran values for AS sampling combined with FS sampling indicated significant positive spatial autocorrelation, except in 2005, which was the year with the highest ratio of FS to AS sampling.

**Table 3:** Global Moran I statistics and relative standard deviation (RSD) for the active surveillance programme 2002 – 2005.

	Fallen Stock		Abattoir Survey		Combined FS & AS	
YEAR	I	RSD	I	RSD	I	RSD
2002	0.07	76.9%	0.16**	80.0%	0.15**	73.8%
2003	0.10*	163.7%	0.37**	70.1%	0.34**	67.9%
2004	0.03	122.1%	0.21**	102.1%	0.16**	83.4%
2005	0.02	76.2%	0.13*	107.6%	0.04	75.5%

Expected Moran's I with no spatial correlation, E [I] = -0.013

* Significant deviation from no spatial correlation (P < 0.05), ** (P < 0.01).

**Figure 5:**
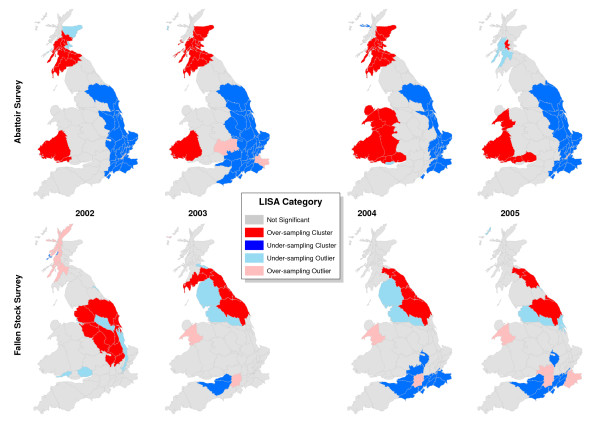
**LISA cluster map indicating significant local spatial correlations**. Colours indicate significant positive (red and blue), negative (pink and pale blue) and not significant (gray) spatial autocorrelation from a Monte-Carlo based test (p < 0.05) on sampling by the abattoir survey (above) and the fallen stock survey (below).

The LISA statistics indicated few exceptions to the overall trend of positive spatial autocorrelation in the abattoir survey (Figure 5). In most years, counties in Wales and Scotland with high sampling rates coincided with neighbouring counties with high sampling rates, while counties in East England with low sampling rates had neighbours with similar sampling rates. Significant contrasts between counties and their neighbours were unusual. In the FS, high sampling in North-east England contrasted with low sampling in North-west England, while relatively high sampling in Wiltshire contrasted with low sampling in much of South England. Gwynedd was a consistently highly sampled county contrasting with the general level of sampling in Wales. The combined FS and AS survey had similar local clustering to the AS survey, although in 2004 and 2005 there were fewer areas with positive local clustering (maps not shown).

### Relationship between sampling and sheep distribution

**Figure 6:**
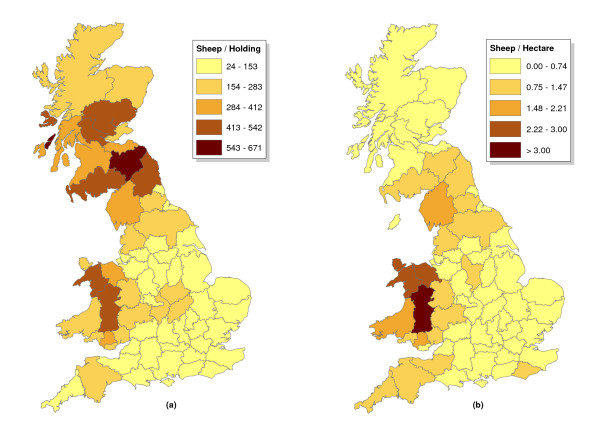
**Maps indicating by county a) the number of sheep per holding and b) the number of sheep ha^-1^**.

The distribution of sampling rate in the abattoir survey seemed correlated with both the geographic distributions of sheep density (sheep/ha) and holding size (sheep/holding) (Figure 3, 6). Indeed the sampling rate in each county during 2002–2005 was strongly correlated with sheep density, holding density and holding size (Table 4). Multivariate logistic regression indicated that the proportion of holdings sampled by county by the AS was strongly dependent on holding density and holding size (Table 5). Coefficients for holding size may appear low, but are multiplied by over 400 sheep per holding in counties with large sheep holdings (Figure 6). In contrast, the proportions of holdings sampled by the fallen stock survey were more weakly correlated with measures of sheep density (Table 4). Only holding size made a marginally significant contribution in a multiple logistic regression (Table 5).

**Table 4:** Correlation matrix for county sampling rates and sheep density measures in active surveillance 2002–2005.

	Holding size	Sheep density	Holding density	AS sample rate	FS sample rate
Holding size	1.000				
Sheep density	0.763	1.000			
Holding density	0.154	0.633	1.000		
AS sample rate	0.446	0.825	0.867	1.000	
FS sample rate	-0.065	0.134	0.329	0.249	1.000

AS is the abattoir survey, FS is the fallen stock survey.

**Table 5:** Coefficients from fitting a GLM regression model for sampling rate to three measures related to sheep density.

Factor/Statistic	Abattoir survey	95% confidence interval	Fallen stock survey	95% confidence interval
Constant	**-2.63**	**-3.05 – -2.20**	**-2.69**	**-3.42 – -1.97**
Holding size (sheep)	**0.0046**	**0.0033 – 0.0059**	**0.0022**	**0.0005 – 0.0043**
Sheep density (ha^-1^)	0.10	-0.15 – 0.36	-0.14	-0.62 – 0.34
Holding density (km^-2^)	**1.42**	**0.62 – 2.22**	-0.61	-2.12 – 0.91
% Deviance from uniform explained	**88.0**		**16.1**	

Bold emphasizes statistically significant values (P < 0.05).

**Figure 7:**
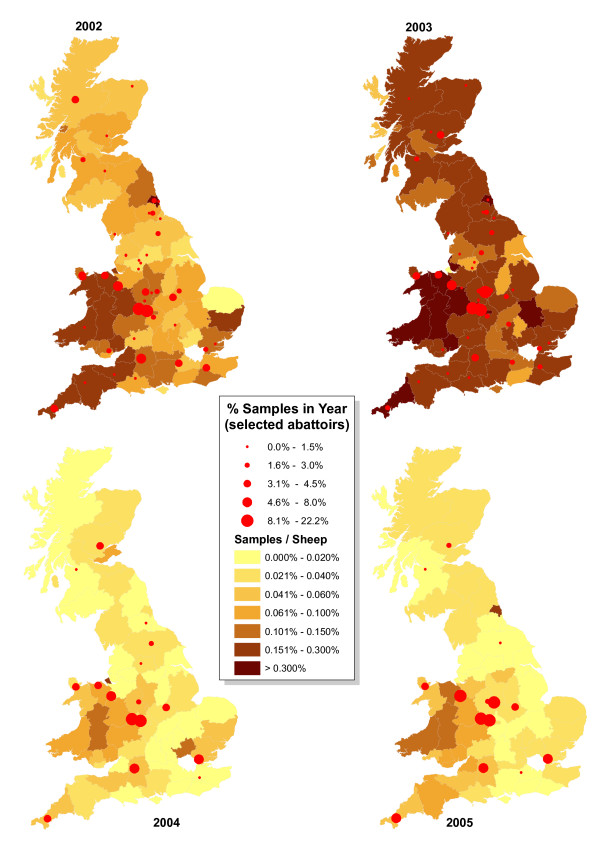
**Location of main abattoirs participating in the abattoir survey each year during 2002–2005, relative to sheep sampling rate**.

Sampling by the abattoir survey expressed as a proportion of county sheep populations was unevenly distributed, with a similar geographic pattern to the distributions of the proportion of holdings sampled and the density of sheep (Figure 7). Thus sheep in counties with dense sheep populations were sampled more heavily than sheep in counties with sparse sheep populations. The overall distribution of sampling had no direct relationship with the locations of abattoirs at which samples were collected (Figure 7). Most abattoirs are not located within areas with dense sheep populations. The distribution of sampling among the largest abattoirs was consistent between years and sampling was dominated by a small number of large abattoirs, e.g. five abattoirs provided over 50% of the samples. This phenomenon was not accidental, because the abattoirs were selected each year to collectively take over 85% of sheep slaughtered over the age of 18 months. Sampling at smaller abattoirs declined as total samples reduced: the number of abattoirs collecting over 10 samples was 45 and 47 in 2002 and 2003, but only 17 and 15 in 2004 and 2005. Nevertheless the relative distribution of sheep sampling in 2004 and 2005 remained roughly similar to 2002 and 2003, despite very few samples being collected at abattoirs in Northern England, suggesting that sheep routinely travel long distances to the large Midlands abattoirs.

## Discussion

There was clear evidence that sampling of sheep holdings by both the abattoir survey and the fallen stock survey was unevenly distributed. Moreover, this uneven sampling apparently affected the distribution of detected cases of scrapie. Apart from the relatively small fallen stock survey in 2002, both spatial distributions were consistent through the years 2002–2005, so that the same regions continued to be over-sampled or under-sampled each year. Although there was some evidence that sampling rates in the fallen stock survey became more uniform during 2003–2005, it is likely that similar spatial distributions will continue if not actively corrected in sympathy with disease occurrence. The consistent relatively low sampling rates in the South-east may deserve especial attention, given the historically high incidence of scrapie as detected by passive surveillance and postal survey in that part of Britain [12,28].

Positive global Moran statistics indicated that the abattoir survey sampling was not only uneven, but was spatially autocorrelated at the county level, suggesting broad regional trends. In contrast, there was weaker evidence of spatial correlation at the county level in the fallen stock survey, and sampling by the fallen stock survey could be negatively correlated in neighbouring counties as well as positively correlated, suggesting that factors influencing fallen stock sampling were more local. Combining the two surveys reduced the contrast between heavily sampled counties and lightly sampled counties, but the combined surveys in 2002–2005 still sampled holdings unevenly across Great Britain in a pattern that was consistent between years and spatially autocorrelated.

In the abattoir survey, the variation of holding sampling between counties was strongly related to aspects of sheep distribution, including sheep density, holding density and holding size. The observed strongest regression was with holding density and holding size, whose product is sheep density. The relationship with sheep distribution was so strong that it must have included much of the spatial correlation in sampling among neighbouring counties. The impact of holding size was expected, because the probability that a sheep randomly selected from the British flock comes from a particular holding will be in proportion to the number of sheep in that holding. However, the additional relationship with holding density cannot be so easily explained. Counties with dense sheep populations were sampled disproportionately, so that individual sheep within holdings in those counties were more likely to be sampled than sheep in counties with sparser populations. The dominance of sampling by a small number of large abattoirs may be an important factor, because economies of scale may encourage large abattoirs to source their sheep directly or indirectly from areas with dense animal populations. Large abattoirs were bound to be selected for sampling, because the survey aimed to potentially sample most sheep over 18 months old. This relationship between sampling and animal population distribution may have widespread significance. Variation in local population density is one of the most fundamental characteristics of most national animal populations worldwide, so over-sampling of locally dense populations may be an issue in many surveys that rely on samples taken from pre-existing gathering points, such as abattoirs.

The evidence presented in this study has two applications: to allow interpretation of case distribution taking account of the sample distribution [4] and to allow design for future sampling, ideally relative to disease occurrence, which would maintain the ability to detect cases as the disease becomes rare. In the latter case, the strong regional trend of the AS would make it easier to adjust its sampling than the FS, which has a much patchier distribution with local contrasts. Del Rio Vilas et al. (2005) [3] reported a large variation in the number of samples from each holding in the FS, which was not necessarily correlated with holding size, and suggested that sampling could be arbitrary rather than random. The large numbers of submissions from some holdings to the FS may even reflect exploitation of the free disposal scheme of carcasses under the FS. Such exploitation may be diluting the high-risk nature of the surveillance stream and reducing the value of this targeted approach [6]. The AS, on the other hand, appeared to have achieved a better control of the number of samples taken per holding. However, since 2005, the number of fallen stock samples per holding has been restricted as much as possible.

There have been previous attempts to study the representativeness of the active surveillance of scrapie in other countries. In France, Morignat et al. (2006) [14] simulated the effects of three biases, namely the lack of random sampling at the abattoir, the presence of spatial heterogeneity in the sampling rate and the use of different diagnostic tests, to assess their impact on the surveillance results. The latter two accounted for significant differences in their results, indicating their importance in the design of the sampling. Lynn et al. (2007) [29] conducted an evaluation of the active scrapie surveillance in the U.S. Spatial unevenness was also evident in their study with large disparities in the sampling proportion between states, but they claimed that the representativeness of their surveillance appeared in general to be fair, although the basis of this evaluation was unclear. They suggested further analysis to define more accurately the adequacy of sampling.

One issue in this present study was the potential impact on the sampling distribution of the nearly 40% of the AS samples and about 25% of the FS samples that could not be traced. A higher proportion of FS samples were traced for several reasons. Their collection locations, which were likely to be their birth locations as well, were known, staff collecting FS had more training and time for recording data than staff at abattoirs, and most FS were collected in 2004 and 2005, when recording standards had improved. Comparison with numbers of holdings recorded in each county by the agricultural survey showed that tracing from Shetland, the Western Isles and Highland was poor, but that tracing from other counties was relatively uniform. Using the traceable holdings as the denominator population partly compensated for remaining differences in tracing between counties.

We went beyond cartographic presentation by testing for evidence of spatial correlation among the sampling rates of neighbouring counties. Within the spatial analysis presented here, Moran's I, as a global measure, was adequate to demonstrate the presence of spatial autocorrelation in the abattoir survey, which distinguished its geographic distribution from the distribution of the fallen stock survey. However, the local LISA measures were also useful in identifying local patterns in the fallen stock survey, while confirming that there were few exceptions to the broad regional trends in the abattoir survey. The weak spatial autocorrelation in the fallen stock survey at the county level, despite the differences in sampling between counties, suggests that further spatial structure could be revealed by spatial analysis at a finer resolution.

A full understanding of the corrections required in the surveys must depend on some understanding of the distribution of scrapie cases. For example, the importance of under-sampling of small flocks due to sampling bias strongly depends on whether sheep in small flocks are more or less likely to have scrapie than sheep in larger flocks. These first steps to identifying the sample distribution have given us the opportunity to investigate such issues, adding to our understanding of the disease epidemiology as well as its surveillance.

## Conclusion

Visualizing the distribution of holdings sampled in the scrapie surveys demonstrated their unevenness at the county level, and that the distribution of sampling differed between the two surveys. The distribution of sampling was positively correlated from year to year, suggesting that uneven sampling will continue unless actively corrected. An alternative to correcting uneven sampling is to take account of the distribution of sampling when analysing survey results, now that we have the information. Combining the two surveys reduced the difference between the most heavily sampled counties and the most lightly sampled counties, but levels of sampling still differed substantially between counties. A large proportion of holdings providing many samples was an issue with the fallen stock survey, which will affect its effectiveness for scrapie surveillance. Initial spatial analysis at the coarse, county level indicated significant spatial autocorrelation of sampling in the abattoir survey. Abattoir survey sampling was strongly positively related to parameters of sheep distribution, including sheep density, holding density and numbers of sheep per holding, so that sheep in counties with high sheep densities were more likely to be sampled. We suggest that this positively density-dependent sampling may be caused by most samples coming from a few, large abattoirs. Modifying the distribution of sampling between abattoirs appears to be the most practicable option to achieve more uniform sampling, so the next step will be more detailed analysis of abattoir catchments.

## Authors' contributions

CB designed the algorithm for tracing birth holdings, initiated the comparison of sampling rates in cartograms and the regression against sheep density, advised on, contributed to and checked analyses, and produced the final version of the manuscript. AC geo-referenced points, prepared maps, carried out most of the geostatistical and local cluster analysis, and drafted the manuscript. KH critically discussed results and conclusions and revised the manuscript. VDR defined the problem, provided epidemiology domain knowledge, critically discussed results and conclusions, and revised the regression model. All authors read and approved the final manuscript.
